# Supplementary material for: Splice-Junction-Based Mapping of Alternative Isoforms in the Human Proteome
Source: Cell Rep. Author manuscript; Available in PMC 2020 Jan 15. (PMC6961840; doi:10.1016/j.celrep.2019.11.026)
Supplement: 2 [file NIHMS1546469-supplement-2.zip › DF1/PXD009737/TestisTrypsin/TestisTrypsin_72_EIF5A_PGDAGASATFPMQCSALR.pdf]

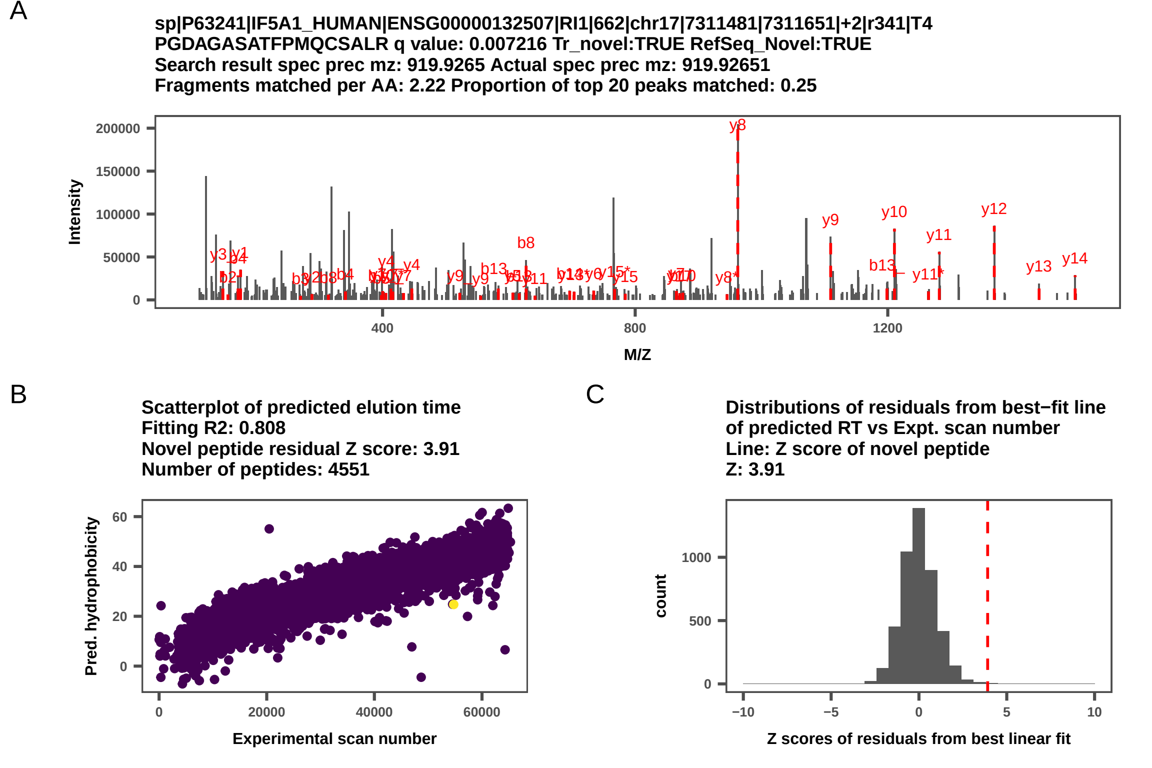
A
sp|P63241|IF5A1_HUMAN|ENSG00000132507|RI1|662|chr17|7311481|7311651|+2|r341|T4
PGDAGASATFPMQCSALR q value: 0.007216 Tr_novel:TRUE RefSeq_Novel:TRUE
Search result spec prec mz: 919.9265 Actual spec prec mz: 919.92651
Fragments matched per AA: 2.22 Proportion of top 20 peaks matched: 0.25
Intensity
200000
150000
100000
50000
0
y8
y9
y10
y11
y12
y13
y14
b8
b13
y11*
y8*
400
800
1200
M/Z
B
Scatterplot of predicted elution time
Fitting R2: 0.808
Novel peptide residual Z score: 3.91
Number of peptides: 4551
Pred. hydrophobicity
60
40
20
0
0
20000
40000
60000
Experimental scan number
C
Distributions of residuals from best−fit line
of predicted RT vs Expt. scan number
Line: Z score of novel peptide
Z: 3.91
count
1000
500
0
−10
−5
0
5
10
Z scores of residuals from best linear fit
